# Recovery from Abobotulinum Toxin A-Induced Paralysis Following Ozone Injection in a Split-Body Rabbit Model

**DOI:** 10.3390/toxins18100453

**Published:** 2026-10-17

**Authors:** Akın Tuna, Ayşenur Botsalı, Mustafa Şen, Okan Ali Aksoy, Özge Banu Öztürk, Ercan Çalışkan

**Affiliations:** 1 Department of Dermatology and Venereology, Gulhane Medical Faculty, University of Health Sciences, 06018 Ankara, Turkey; 2 Department of Animal Experiments and Research Centre, Gulhane Training and Research Hospital, University of Health Sciences, 06018 Ankara, Turkey; 3 Private Clinic, 06810 Ankara, Turkey

**Keywords:** botulinum toxin, complication management, ozone injection, functional recovery, ozone therapy, rabbit model

## Abstract

Although previous studies have demonstrated that ozone gas can inactivate botulinum toxin in liquid solutions, evidence regarding its effects under *in vivo* conditions remains limited. This exploratory study investigated whether local ozone injection is associated with earlier functional recovery from abobotulinum toxin A-induced muscle paralysis in a rabbit model. Three New Zealand White rabbits received bilateral intramuscular injections of 15 U abobotulinum toxin A into the anterior auricular muscles. After maximal paralysis was established, the right anterior auricular muscle received 1 mL ozone gas at a concentration of 10 µg/mL twice weekly for two weeks (four sessions in total), while the left side received room air injections as the concurrent control. Ear positions were assessed by twice-weekly imaging using a four-grade visual paralysis scale (Grade 0–III) until baseline position was restored. All ozone-treated ears improved from Grade III to Grade II by day 7 and returned to their baseline positions (Grade 0) by day 10, remaining at Grade 0 thereafter. In contrast, the room-air control ears remained at Grade III through week 3, showed progressive improvement during weeks 4 and 5, and returned to Grade 0 by week 6. Previous findings using the same experimental model without additional intervention reported persistence of paralysis for approximately 16 weeks. This study provides the first *in vivo* experimental evidence suggesting that local ozone administration may be associated with earlier functional recovery from abobotulinum toxin A-induced paralysis. However, larger, randomized, and objectively assessed studies are required to confirm this observation and clarify the underlying mechanisms.

## 1. Introduction

Botulinum toxin (BoNT) induces temporary chemical denervation and muscle paralysis through the inhibition of acetylcholine release at the neuromuscular junction, specifically at the peripheral motor neuron terminals. In the field of aesthetic dermatology, the administration of botulinum toxin (BoNT) requires precise technique and accurate dosing. Deviations from established injection protocols can lead to unintended diffusion of the toxin into adjacent musculature, thereby increasing the risk of complications such as blepharoptosis, diplopia, ectropion, facial asymmetry, and asymmetric motor activity of mimetic muscles [1].

The frequency of complications decreases with expertise, but does not disappear entirely. The estimated incidence of BoNT-A-induced blepharoptosis is almost 1% among experienced injectors compared to 5.4% among inexperienced injectors [2]. The aesthetic applications of Botulinum Toxin (BoNT) have broadened within the field of cosmetic medicine, resulting in a concomitant increase in the number of physicians administering BoNT injections. This escalation has been associated with a rise in the incidence of complications related to its use.

Although complications associated with botulinum toxin injections are generally reversible, the functional and cosmetic impairments can be problematic and frequently necessitate prompt intervention. In such cases, patients seek aesthetic enhancements but are often met with significant disappointment.

Presently, there is no universally accepted or standardized treatment protocol for managing such adverse effects. Oxymetazoline HCl 0.1% eye drop is the only FDA-approved drug for the treatment of BoNT-related blepharoptosis [3]. Numerous case reports and series have documented various modalities, and given the urgency associated with reversing muscle paralysis, clinicians are increasingly exploring synergistic combination therapies [4,5,6].

Previous *in vivo* studies have suggested that ozone may modulate several biological processes involved in tissue repair and recovery, including microvascular responses, angiogenesis, fibroplasia, and oxidative and inflammatory pathways [7,8,9]. These experimental findings provide a biological rationale for investigating ozone in conditions involving tissue and neuromuscular recovery. Although previous studies have demonstrated the ability of ozone gas to inactivate botulinum toxin in liquid solutions, evidence regarding its effects under *in vivo* conditions remains limited. Given its potent oxidative properties, ozone may interact with botulinum toxin or influence local biological processes involved in neuromuscular recovery, potentially facilitating recovery from toxin-induced muscle paralysis [10,11,12].

An earlier study on the bioactivity of abobotulinum toxin-A in a rabbit model demonstrated that the anterior auricular muscle paralysis related to the toxin resolved within 16 weeks without intervention [13]. Building on this model, we hypothesized that targeted intramuscular ozone gas injections would accelerate functional recovery from botulinum toxin-induced muscle paralysis *in vivo*. Accordingly, the present study investigated the effect of ozone injection on recovery from abobotulinum toxin A-induced anterior auricular muscle paralysis using a split-body rabbit model. From a dermatological perspective, this experimental approach may provide a basis for exploring strategies to shorten the duration of unintended muscle weakness following botulinum toxin administration.

## 2. Results

**Figure 1:**
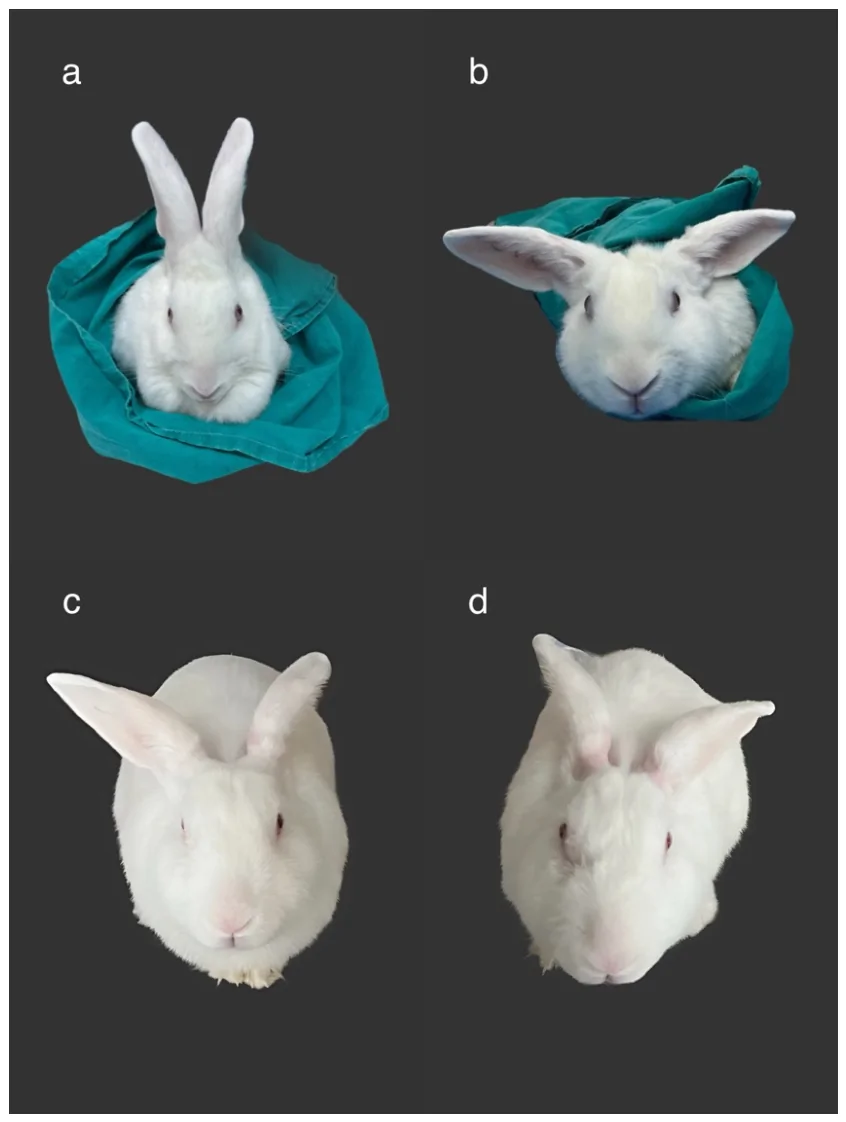
(**a**) Baseline position of the ears. (**b**) Two weeks post-injection of botulinum toxin, demonstrating grade III paralysis in both ears. (**c**) Within the first week after starting ozone injections, all three treated right ears showed an improvement exceeding 30° (Grade II) in the ozone-treated right ears. (**d**) By day 10, the ozone-treated right ears showed complete recovery (Grade 0), whereas the left ears injected with room air showed no positional improvement.

Maximal muscle paralysis was observed on the 14th day post-injection of Abobotulinum toxin-A, corresponding to grade III severity, and was consistently observed across all six injected ears (Figure 1).

The alterations in muscle paralysis grades at the respective injection sites following the anti-paralysis interventions (ozone versus room air injections) are detailed in Table 1.

**Figure 2:**
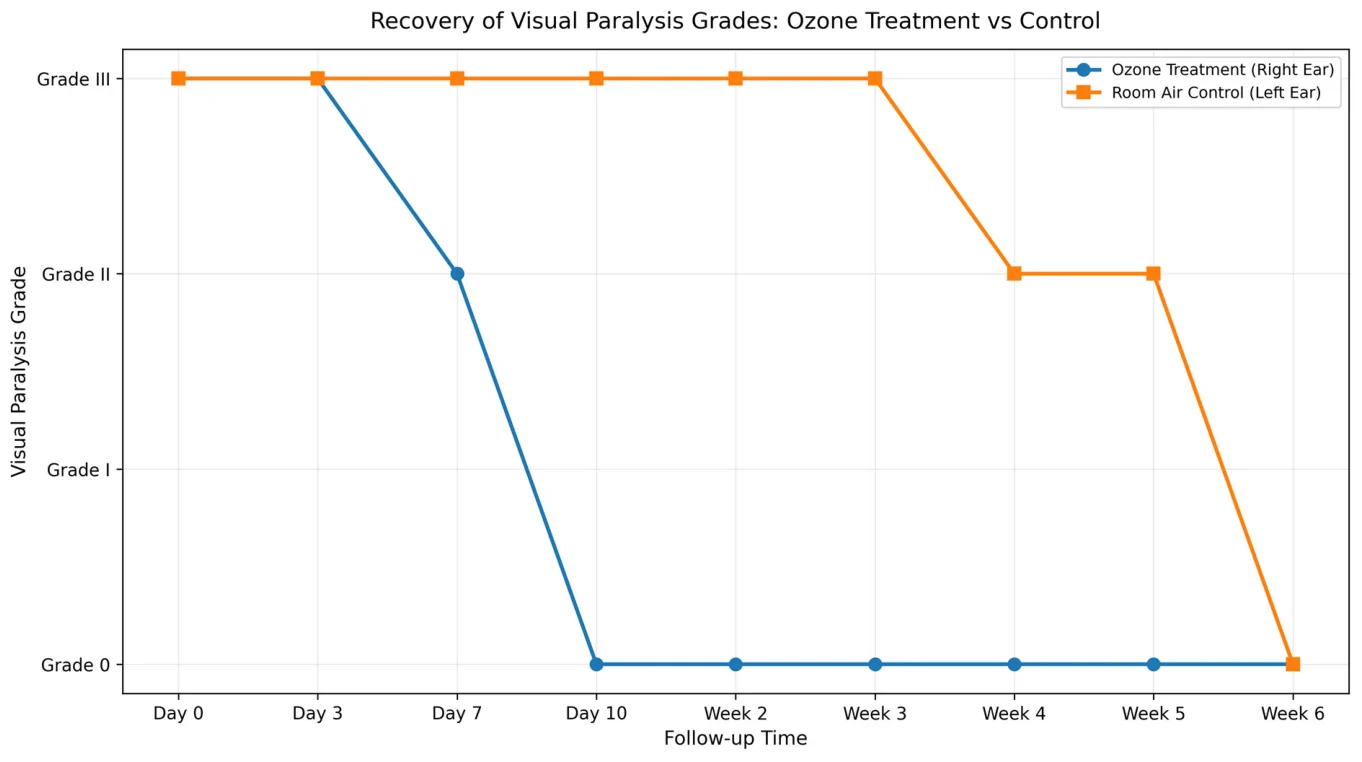
Muscle paralysis grade recovery over 6-week follow-up period.

By day 7 following the initiation of ozone injections, all three ozone-treated right ears had improved from Grade III to Grade II, and by day 10, all had returned to their baseline positions (Grade 0). In contrast, the room-air control ears remained at Grade III throughout the intervention period and through week 3. All injections were discontinued after completion of the two-week treatment period. During twice-weekly follow-up, partial recovery of the control ears became evident at week 4, with scores ranging from Grade II to Grade III, followed by further improvement at week 5 (Grade I–II). By week 6, all control ears had returned to their baseline positions (Grade 0) (Figure 2). For contextual comparison, these findings were considered alongside a previous study using the same rabbit model, in which five rabbits receiving an equivalent dose of abobotulinum toxin A were followed without additional intervention and paralysis persisted for approximately 16 weeks.

## 3. Discussion

Various therapeutic approaches have been explored for managing eyelid and brow ptosis induced by botulinum toxin, a potential adverse effect associated with cosmetic botulinum toxin interventions. The onset of symptoms typically occurs within 3 to 14 days post-injection, presenting either unilaterally or bilaterally, and generally resolves within a 3- to 4-week period. Nonetheless, existing literature also documents cases in which ptosis persisted for approximately 6 to 13 weeks following onset [14]. Variables such as muscle size and the dose of botulinum toxin may influence the duration of both therapeutic effects and complications. In the present study, a split-body design was employed to allow within-animal comparison between the ozone-treated and room-air control sides. When evaluating the relative size of the rabbit’s anterior auricular muscle in comparison to the human levator palpebrae muscle, and considering their respective mass and functional roles, it can be argued that the findings obtained from this animal model may have limited applicability when extrapolated to clinical scenarios. Consequently, the authors propose that a more rapid onset of effect may be achievable in cases of blepharoptosis.

In the present study, the right ears of the animals returned to their original pre-injection position by day 10, whereas recovery at the room air-treated sites was observed at six weeks. The marked difference between the ozone-treated sites (10 days) and room air-treated sites (6 weeks) suggests an association between localized ozone administration and earlier functional recovery from botulinum toxin-induced paralysis. However, these findings do not establish direct neutralization or degradation of botulinum toxin bioactivity. Interestingly, recovery at the room air-treated sites was also faster than that reported in a previous study using an identical model without additional intervention, in which paralysis persisted for approximately 16 weeks. This unexpectedly shorter recovery period in the room air-treated sites could potentially reflect factors such as local diffusion of ozone to adjacent tissues or mechanical effects associated with repeated twice-weekly punctures; however, the extent to which these factors may have contributed to the observed recovery cannot be established based on the present study design.

Current perspectives on the recovery from botulinum toxin (BoNT)-induced denervation suggest that it may result either from the gradual regeneration of nerve terminals as part of the inherent wound-healing processes activated by the puncture injury or from the diminution of the neurotoxin concentration through dilution.

Rullan et al. reported a case series comprising twenty-two patients who developed brow ptosis subsequent to botulinum toxin administration [5]. These patients were subsequently managed with intramuscular injections of bacteriostatic saline as a corrective intervention. The bacteriostatic saline was administered in weekly sessions, precisely targeted above the region of ptosis and into the immobilized segments of the lower frontalis and medial corrugator muscles. Importantly, a significant proportion of the cohort (86.4%) experienced resolution of ptosis following a single injection, whereas 9.1% required two injections, and 4.5% necessitated three injections. To elucidate the specific role of bacteriostatic saline, a control cohort comprising four patients undergoing botulinum toxin (BoNT) injections for the treatment of forehead and glabellar lines was incorporated into the study design [5]. At two weeks post-injection—when the therapeutic efficacy of BoNT was evident—the contralateral (left) sites served as internal controls by remaining untreated. Conversely, the ipsilateral (right) sites received injections of either 1 mL of bacteriostatic saline (*n* = 2), sterile water (*n* = 1), or normal saline (*n* = 1). The findings demonstrated that only the sites injected with bacteriostatic saline exhibited detectable muscular activity, in contrast to those treated with sterile water or normal saline. The authors suggest that the observed efficacy of bacteriostatic saline is unlikely to be attributable solely to mechanical disruption caused by needle puncture. Instead, they hypothesize that the benzyl alcohol (BnOH) component may play a significant role. BnOH, a nonpolar solvent, is postulated to facilitate the dissolution of nonpolar substances such as lipids, which constitute fundamental components of cellular membranes. This dissolution process may enhance membrane permeability and disrupt endosomal transport mechanisms [5].

Therapeutic strategies used to manage complications of botulinum toxin treatment may improve clinical symptoms through mechanisms that do not directly affect the toxin itself. For instance, ophthalmic pharmacological agents such as oxymetazoline and apraclonidine exert their effects by stimulating the superior tarsal muscles through the activation of the sympathetic nervous system. Nonetheless, the clinical efficacy of these interventions remains generally limited, and among the spectrum of complications associated with botulinum toxin, such agents are exclusively indicated for the management of blepharoptosis [15,16]. A documented case involved a 76-year-old female patient with a history of glaucoma and hemifacial spasms who received botulinum toxin type A (BoNT-A) injections. Subsequently, approximately two weeks post-procedure, the patient developed left-sided blepharoptosis accompanied by notable visual impairment. The condition was managed with the administration of 0.5% apraclonidine eye drops, with immediate clinical response observed. Follow-up visual assessments conducted at 30 and 45 days post-injection indicated an improvement in blepharoptosis, although complete resolution was not achieved [6].

An alternative therapeutic approach involves modulating muscle activity by targeting the antagonistic muscles through transdermal injections of botulinum toxin [17]. Specifically, injections are administered to the pre-tarsal orbicularis oculi muscle to ameliorate blepharoptosis and eyelid asymmetry. According to Mustak et al.’s protocol, 3 units of botulinum toxin type A (BoNT-A) are injected 2 mm above the lash line [17]. Follow-up assessments conducted between 6 and 12 weeks post-injection demonstrated an increase in marginal reflex distance (MRD) of approximately 0.9 mm [17].

Ozone injections are postulated to induce vasodilation of capillaries, enhance the oxygen-carrying capacity of erythrocytes, and upregulate the expression of local growth factors, thereby potentially facilitating cellular regeneration [18,19]. Additionally, owing to its strong oxidative properties, ozone has been reported to interact with and alter various biological molecules and toxins in liquid media, including botulinum toxin. This process presumably involves the oxidation of amino acids and sulfhydryl groups within peptides, proteins, and enzymes, leading to the formation of smaller peptide fragments [20].

The present study utilized a small sample size (*n* = 3) and a fixed split-body design, with the right anterior auricular muscle consistently assigned to ozone treatment and the left side to room-air control. Side allocation was not randomized, which represents a methodological limitation of the study. Despite these limitations, a marked difference in recovery time was observed: return to baseline ear position occurred within 10 days on the ozone-treated side, compared with 6 weeks on the room-air control side. Notably, the 6-week recovery observed in the control ears was itself shorter than the approximately 16-week duration reported in previous studies using the same animal model without intervention. Although these findings should be interpreted cautiously, the pronounced difference in recovery trajectories supports further investigation of ozone as a potential modulator of recovery from botulinum toxin-induced paralysis.

## 4. Conclusions

This preliminary exploratory study suggests that local ozone gas injection may be associated with earlier functional recovery from botulinum toxin-induced muscle paralysis, with complete recovery observed within 10 days on the ozone-treated side compared with 6 weeks on the room-air control side. These findings should nevertheless be interpreted within the context of the exploratory study design, which included a small sample size, fixed side allocation, no blinding or concurrent untreated control group, and no objective functional or inferential statistical assessments. Prospective randomized controlled studies with larger sample sizes and objective outcome measures are warranted to validate these preliminary findings and further investigate the mechanisms underlying the observed recovery following ozone injection.

## 5. Methods

This study was reviewed and approved by Gulhane Animal Experiments Local Ethics Committee (protocol number: 24/12, dated 25 April 2024), and conducted in accordance with national and international guidelines for the care and use of laboratory animals.

### 5.1. Study Design

The trial was conducted from 15 October to 15 December 2024. OAA supervised all procedures as the veterinarian physician. The study involved three young male New Zealand White rabbits, each weighing between 3.5 and 4.5 kg. The subjects were housed individually under standardized conditions and were provided with ad libitum access to food and water.

#### 5.1.1. Anesthesia

Given the minimally invasive nature of the procedures and the inherent risks associated with general anesthesia—particularly hypothermia, which can be lethal in rabbit subjects—the injections were conducted without the administration of anesthetic agents. Instead, subjects were immobilized through physical restraint, achieved by wrapping them in cloth to expose only the target injection site. The animals appeared to tolerate the procedure well under these conditions.

#### 5.1.2. Injection Point and Technique

Both ears of the three rabbits were shaved before the injections. The anterior auricular muscle (AAM) was designated as the target site for all injections owing to its clearly delineated origin and insertion points, which are readily palpable. This anatomical clarity enhanced the precision of injections and ensured reliable evaluation of muscle function.

Each anterior auricular muscle (mAA) of three male New Zealand White rabbits received 15 units of Abobotulinum toxin A through a single puncture to create the muscle paralysis model. The model was deemed valid if a Grade III analysis was observed at the injected ear. At the time of maximal paralysis, the right mAA of each animal received a 1 mL intramuscular injection of ozone (O3) at a concentration of 10 µg/mL using a 27-gauge needle, administered twice weekly over two weeks, not exceeding a total number of four treatment sessions. Concurrently, room air was injected into the left mAA to serve as a control for comparative analysis. Ozone was generated using the Salutem Plus® Revision 3 device (Has Medikal, Medical and Electronic Devices, İzmir, Turkey), which was used consistently throughout the study to ensure precise and controlled ozone delivery. The ozone concentration of 10 µg/mL was selected based on previous experimental and clinical studies employing low-concentration ozone. Low-dose ozone exposure has been proposed to induce a mild and controlled oxidative stimulus that may trigger adaptive antioxidant and cytoprotective responses, whereas higher concentrations may result in excessive oxidative stress. A concentration of 10 µg/mL has been used in several experimental and clinical settings, including peripheral neural, musculoskeletal, and intra-articular applications, supporting its selection as a low-dose concentration for the present study [13,21].

#### 5.1.3. Photography and Follow-Up Period

Ear positions were assessed through visual analysis of sequential photographs. Images were captured twice weekly using a digital camera, commencing at baseline and continuing until the sixth week post-final injection.

### 5.2. Outcome Measures

**Figure 3:**
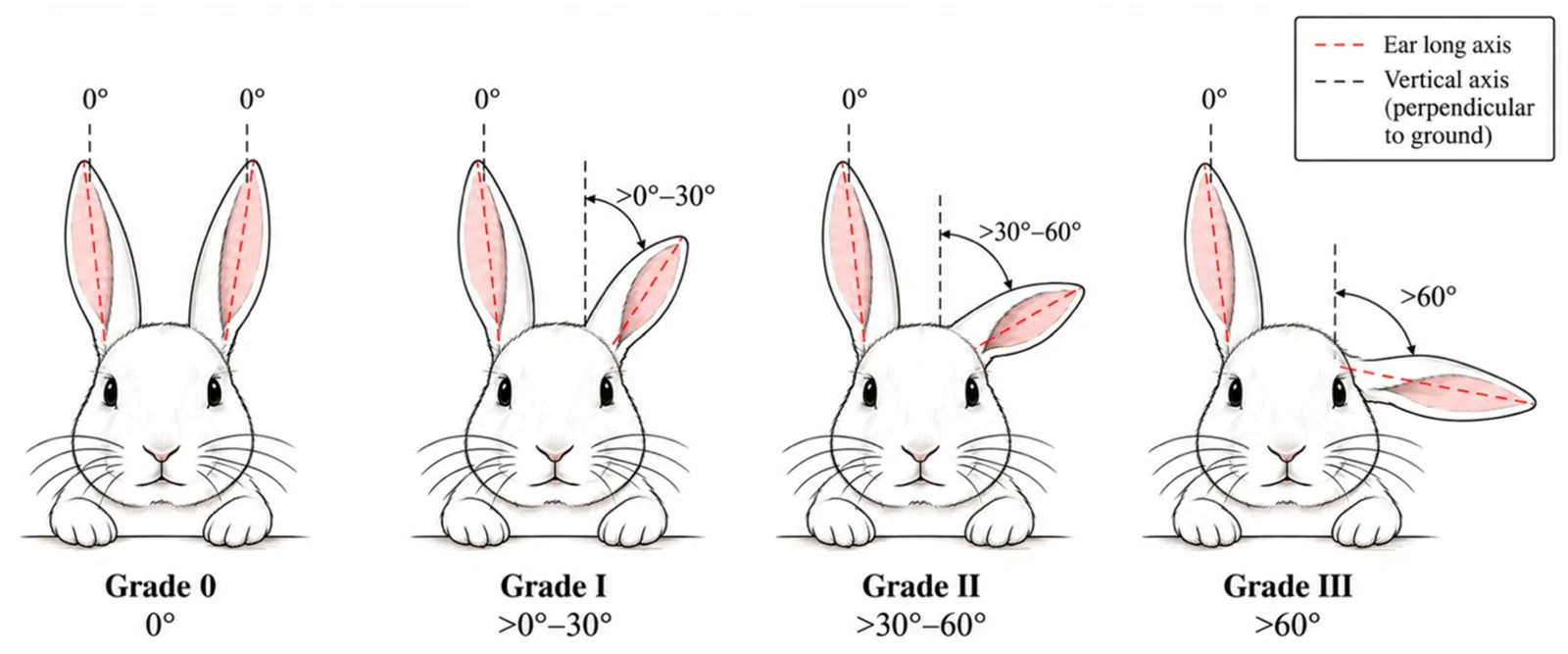
Static grading system (Grades 0–III) for rabbit auricular position. The angle was measured between the longitudinal axis of the ear and a vertical reference axis perpendicular to the ground. Grade 0 indicates no paralysis, with the ear maintaining its baseline position (0°). Grade I represents mild paralysis, characterized by a downward displacement of >0–30°. Grade II represents moderate paralysis, with a downward displacement of >30–60°. Grade III represents severe paralysis, characterized by a downward displacement exceeding 60°.

The severity of paralysis was assessed using a visual scoring system based on the ‘static grading system” established by Jabor et al. [22]. Based on this scoring system, four discrete grades were defined: Grade 0 indicated no paralysis, with the ear maintaining its baseline position; Grade I indicated mild paralysis, characterized by a downward displacement of >0° to 30°; Grade II indicated moderate paralysis, with a downward displacement of >30° to 60°; and Grade III indicated severe paralysis, characterized by a downward displacement exceeding 60° (Figure 3). The contralateral left ear served as the room-air control side throughout the study.

To ensure adherence to ethical standards and reduce animal utilization, the experimental results were benchmarked against data from a prior study utilizing an identical model. In the referenced study, the duration of paralysis induced by Abobotulinum toxin A was assessed without the application of additional interventions [13].

## Figures and Tables

**Table 1:** Distribution of visual paralysis scores throughout the study. Grade 0: no paralysis, with maintenance of the baseline ear position; Grade I: mild paralysis (>0–30° downward displacement); Grade II: moderate paralysis (>30–60° downward displacement); Grade III: severe paralysis (>60° downward displacement).

Visual Paralysis Scores	I		II		III	
	Left	Right	Left	Right	Left	Right
Day 0	Grade 3	Grade 3	Grade 3	Grade 3	Grade 3	Grade 3
Day 3	Grade 3	Grade 3	Grade 3	Grade 3	Grade 3	Grade 3
Day 7	Grade 3	Grade 2	Grade 3	Grade 2	Grade 3	Grade 2
Day 10	Grade 3	Grade 0	Grade 3	Grade 0	Grade 3	Grade 0
Week 2	Grade 3	Grade 0	Grade 3	Grade 0	Grade 3	Grade 0
Week 3	Grade 3	Grade 0	Grade 3	Grade 0	Grade 3	Grade 0
Week 4	Grade 3	Grade 0	Grade 2	Grade 0	Grade 2	Grade 0
Week 5	Grade 2	Grade 0	Grade 2	Grade 0	Grade 1	Grade 0
Week 6	Grade 0	Grade 0	Grade 0	Grade 0	Grade 0	Grade 0

## Data Availability

The original contributions presented in this study are included in the article. Further inquiries can be directed to the corresponding author.
